# Developing Seasonal Ammonia Emission Estimates with an Inverse Modeling Technique

**DOI:** 10.1100/tsw.2001.312

**Published:** 2001-11-21

**Authors:** Alice B. Gilliland, Robin L. Dennis, Shawn J. Roselle, Thomas E. Pierce, Lucille E. Bender

**Affiliations:** NOAA Air Resources Laboratory, Research Triangle Park, NC 27709, USA

**Keywords:** ammonia emissions, emissions uncertainty and evaluation, air quality modeling, inverse modeling

## Abstract

Significant uncertainty exists in magnitude and variability of ammonia (NH_3_) emissions, which are needed for air quality modeling of aerosols and deposition of nitrogen compounds. Approximately 85% of NH_3_ emissions are estimated to come from agricultural nonpoint sources. We suspect a strong seasonal pattern in NH_3_ emissions; however, current NH_3_ emission inventories lack intra-annual variability. Annually averaged NH_3_ emissions could significantly affect model-predicted concentrations and wet and dry deposition of nitrogen-containing compounds. We apply a Kalman filter inverse modeling technique to deduce monthly NH3 emissions for the eastern U.S. Final products of this research will include monthly emissions estimates from each season. Results for January and June 1990 are currently available and are presented here. The U.S. Environmental Protection Agency (USEPA) Community Multiscale Air Quality (CMAQ) model and ammonium (NH_4_) wet concentration data from the National Atmospheric Deposition Program (NADP) network are used. The inverse modeling technique estimates the emission adjustments that provide optimal modeled results with respect to wet NH_4_ concentrations, observational data error, and emission uncertainty. Our results suggest that annual average NH_3_ emissions estimates should be decreased by 64% for January 1990 and increased by 25% for June 1990. These results illustrate the strong differences that are anticipated for NH_3_ emissions.

## INTRODUCTION

Ammonia ($NH_3$) emissions are a vital input for modeling regional patterns of nutrient deposition, visibility, fine particulates, and acid precipitation. According to the U.S. Environmental Protection Agency (USEPA) National Air Pollutants Emissions Trends report[1], $NH_3$ emissions come predominantly from agricultural sources, primarily from livestock (Table 1). Most emission inventories are currently limited to annual total estimates of $NH_3$ emissions[1,2]. We qualitatively know that livestock agriculture and fertilizer emissions vary seasonally according to meteorological conditions and agricultural practices[2]. For example, $NH_3$ is emitted into the air from livestock agriculture by the volatilization of $NH_3$, which is a function of the animal waste temperature[3]. However, insufficient information exists to

**TABLE 1**
**National Emission Inventory $NH_3$ Emission Estimates[1] by Major Sources (thousand short tons)**

| Source Type | 1990 | 1991 | 1992 | 1993 | 1994 | 1995 |
|---|---|---|---|---|---|---|
| Livestock agriculture | 3307 | 3324 | 3341 | 3370 | 3399 | 3427 |
| Fertilizer application | 420 | 446 | 473 | 499 | 525 | 551 |
| Other (industrial sources, mobile) | 604 | 620 | 635 | 652 | 665 | 687 |
| **Total (All Sources)** | **4331** | **4390** | **4449** | **4521** | **4589** | **4665** |

deduce the seasonal variability of these emissions quantitatively for a regional scale domain.

A seasonal factor is needed for these $NH_3$ inventories for use in air quality models because $NH_3$ has a significant role in tropospheric chemistry[2,4]. Pierce and Bender[5] roughly derived seasonal allocation factors for a U.S. $NH_3$ emission inventory using information available about the emission sources. Allocation factors were estimated using patterns of crop planting and fertilizer application and a literature-based analysis of livestock emissions. The resulting seasonal allocation factors were highest during the summer (48% increase) and lowest during the winter (36% decrease), as would be expected. These minimum and maximum allocation factors are supported by concentrations of $NH_3$ fluxes measured at a hog waste lagoon in North Carolina, where the largest fluxes were observed during the summer months and the lowest fluxes during the winter months[3]. Ambient concentrations in two European field studies also show strong seasonal variability, where $NH_3$ concentrations are higher in summer than in winter[6,7]

The purpose of this study is to quantify seasonal variations in $NH_3$ emission estimates on a regional scale for the Eastern U.S. as an inverse problem. Observed and modeled data are used in an optimization formula to estimate emissions that cannot been directly measured. Inverse modeling techniques have been used in a variety of applications to estimate quantities that are not directly available, including estimation of emissions[8,9,10]. After describing the inverse modeling methodology, results for a winter and summer 1990 month will be presented herein, and our ongoing work and future plans will be summarized.

## METHODS

### Inverse Modeling Technique

The adaptive-iterative Discrete Kalman Filter (DKF)[8] is a time-independent version of the sequential DKF that has been used for time-varying emissions[9,10]. The adaptive-iterative DKF has been used in several inverse modeling studies to deduce time-varying isoprene[11] and carbon monoxide[12,13] emissions. A synopsis of the technique is described below. For more details on implementing this inverse modeling technique, refer to Haas-Laursen et al.[8] and Gilliland and Abbitt[13]. The adaptive-iterative DKF formulation is summarized in Eq. 1:

$$\begin{bmatrix} \hat{E}_{t,i+1}(1) \\ \vdots \\ \hat{E}_{t,i+1}(m) \end{bmatrix} = \begin{bmatrix} \hat{E}_{t,i}(1) \\ \vdots \\ \hat{E}_{t,i}(m) \end{bmatrix} + \left\{ \begin{bmatrix} G_{t,i}(1,1) & \dots & G_{t,i}(1,n) \\ \vdots & \ddots & \vdots \\ G_{t,i}(m,1) & \dots & G_{t,i}(m,n) \end{bmatrix} \left( \begin{bmatrix} \chi_t^{obs}(1) \\ \vdots \\ \chi_t^{obs}(n) \end{bmatrix} - \begin{bmatrix} \chi_{t,i}^{\mathrm{mod}}(1) \\ \vdots \\ \chi_{t,i}^{\mathrm{mod}}(n) \end{bmatrix} \right) \right\} \quad (1)$$

or without matrices written out:

$$\hat{E}_{t,i+1} = \hat{E}_{t,i} + G_{t,i}\left(\chi_t^{obs} - \chi_{t,i}^{\mathrm{mod}}\right),$$

where $\hat{E}_{t,i+1}$ $(m\times1)$ represents the adjusted value for the integrated emissions for time $t$ and the next iteration $i+1$ of the air quality model, and $m$ denotes the number of source regions. $\hat{E}_{t,i}$ $(m \times 1)$ represents the integrated emissions for the previous model iteration $i$. In this application, $t$ = 4 weeks or approximately 1 month. As the name implies, the gain matrix $G_{t,i}$ determines the sensitivity of the final emission adjustment to the differences between the observed and modeled concentrations, $\chi_t^{obs}(n\times1)$ and $\chi_{t,i}^{mod}(n\times1)$. The array dimension $n$ denotes the number of observations. The optimal gain matrix $G_{t,i}$ is given by

$$G_{t,i} = C_{t,i}P_t^T\left(P_tC_{t,i}P_t^T + N_t\right)^{-1} \quad (2)$$

where $N_t$ is the variance of error in the observed concentrations, $P_t$ is the Jacobian of the change in concentration with respect to emissions,

$$\left(P_t = \frac{\partial f}{\partial E_t}\right),$$

$f(\,)$ is the function that calculates $(\chi_{t+1} - \chi_t)$ (i.e., the chemical transport model), and $C_{t,i}$ is the variance of error in the previous emissions estimate $\hat{E}_{t,i}$.

Eq. 1 assumes that the modeled and observed concentrations at the previous time step are equal[13], which can be an issue when considering initial conditions in air quality models. Since we are using monthly time increments $t$ and our observational data are derived from accumulated wet deposition during the model simulation, the agreement of initial conditions is insignificant in this case.

Approaches for quantifying $P_t$ and $C_{t,i}$ are similar to past studies using this method[8,11,12,13]. Specifically, the Jacobian matrix $P_t$ is quantified here in a brute-force fashion in that two parallel simulations are performed for the time increment $t$ where the only difference is a 10% change in emissions. Because the initial concentrations are equal in the two parallel simulations, the Jacobian reduces to

$$P_t = \frac{\partial f}{\partial E_t} = \frac{\partial \chi_t}{\partial E_t}.$$

The initial value for $C_{t,0}$ is set to equal $50 \times \hat{E}_{t,0}$ as an arbitrarily large number. After $G_{t,i}$ and $\hat{E}_{t,i+1}$ are calculated, we adjust the emission field $\hat{\varepsilon}_{t,i}$ to

$$\hat{\varepsilon}_{t,i+1} = \left(\frac{\hat{E}_{t,i+1}}{\hat{E}_{t,i}}\right)\hat{\varepsilon}_{t,i} = \left(\frac{\hat{E}_{t,i+1}}{\hat{E}_{t,0}}\right)\hat{\varepsilon}_{t,0} \qquad (3)$$

for each source region $m$. $\hat{\varepsilon}_{t,0}$ represents our best initial guess in emissions and is typically based on existing emission inventories[1]. Sensitivity tests performed by Gilliland and Abbitt[13] showed results are quite insensitive to the initial emission guess. The air quality model simulation is then repeated for time increment $t$. $C_{t,i}$ is then updated for the next iteration $i$+1:

$$C_{t,i+1} = C_{t,i} - G_{t,i} P_t C_{t,i}, \qquad (4)$$

and Eq. 1 and 2 are repeated. This process continues through additional iterations until the final emission adjustment for that time increment is sufficiently small, defined in this study as ≤1% of the initial emissions estimate $\hat{E}_{t,0}$. The number of iterations required to adjust the initial emission varies among applications.

Based on these equations, it is clear that the methodology relies on an assumption that the differences in observed and modeled concentrations are caused by emission misspecifications. Model uncertainty can be introduced into the application through the noise matrix $N_t$[8]; however, quantifying this uncertainty is not straightforward, particularly for air quality models. The uncertainty in $NH_3$ emissions is very large compared to the general uncertainty in the model structure; therefore, model uncertainty is not quantified in this application. To test the rigor of the results, model outputs for other related chemical species were compared against data with favorable results. This comparison will not be included explicitly in this manuscript due to space limitations; however, it will be presented in a later paper.

In past studies where an air quality model was used[11, 12,13], only one source region $m$ was defined for this methodology. One reason for defining only one source region is that the DKF technique as described above assumes that the emission errors are Gaussian white noise. This assumption could be incorrect when multiple source regions $m$ rely on the same raw data, formulas, and emission factors, because the emission uncertainties in the source regions would not be independent or uncorrelated. Other pitfalls for defining multiple source regions include situations where monitored data are not evenly distributed and a source region may not have much data or where a source region is affected largely by boundary conditions. To avoid these types of pitfalls and take a simpler approach initially, we will assume that there is only one source region $m$. The implication is that the entire emission field for all sources will be adjusted by a single factor, leaving the spatial distribution of emissions unchanged. In the Results section we will examine the results to see if spatial biases exist. Depending on these results, the methodology will then be refined to address spatial biases and individual source types in more detail.

Pseudodata or twin-experiment tests[8,9,13] were performed to test the approach described above. These tests use model-generated data as reference observations or pseudodata in Eq. 1. Another simulation is then performed using perturbed or modified emissions. If the inverse modeling technique is applied correctly, adjusted emissions should equal approximately the original emissions that were used to produce the reference observations. The pseudodata tests were successful, thereby confirming that the methodology was applied correctly and that the application was suitable for the technique. Therefore, we could proceed using real observational data.

## Air Quality Model and Observational Data

The USEPA Models-3 Community Multiscale Air Quality (CMAQ) model[14,15,16] is used in this study to generate the model data $\chi_{t,i}^{mod}$. CMAQ is an Eulerian air quality model that was developed to simulate $O_3$, acidic deposition, and aerosol chemistry for urban- to regional-scale domains. For this study, CMAQ is configured with 21 tropospheric layers, a horizontal grid resolution of 36 km, and the RADM2[17] chemical mechanism.

The Fifth Generation Penn State/NCAR Mesoscale Model (MM5) is used to generate the meteorology input data fields for CMAQ[18,19] Emissions fields for all chemical species were produced based on the National Emissions Inventory (NEI)[1], Mobile 5a[20] for mobile emissions, and BEIS2[21] for biogenic emissions. The NEI provides county-scale $NH_3$ emissions data that are processed to develop a gridded emission field for CMAQ. The NEI $NH_3$ emissions data are total annual values (Table 1). Therefore, the initial $NH_3$ emission fields used in these simulations have no seasonality, and the DKF inverse modeling approach will be used to estimate emissions for the specific month.

The NEI inventory, as shown in Table 1, has been updated since these simulations were developed. For this reason, emissions used in these simulations are approximately 7% lower than the current NEI inventory. These differences exist because mobile emissions were not included in the previous inventory and because the area source emissions were 3% lower than current estimates. Mobile emissions account for only about 4% of the total $NH_3$ emissions; however, they may influence specific areas significantly. Therefore, we plan to test the sensitivity of our results to the new emissions inventory as an extension of this research.

To estimate $NH_3$ emissions for each month considered, we will compare $NH_4^+$ wet concentration (mg/l) rather than $NH_4^+$ wet deposition (kg/ha) and apply the DKF. An advantage to using wet concentrations is the consideration of the concentration within the rainwater rather than the total amount deposited, which can help to address differences between observed and modeled precipitation. Alternately, using wet concentrations can ignore the effect of diluting the modeled concentrations when precipitation is over-predicted; however, predictions for these simulations tended to under-predict rather than over-predict precipitation. Theoretically, ambient concentrations of $NH_3$ could be used in this application; however, there are no continuous networks collecting ambient $NH_3$ concentration data, while extensive wet deposition data are available from the National Acid Deposition Program (NADP)[20].

We used $NH_4^+$ data collected by the NADP network[22] on a weekly sampling frequency. We focused on January and June 1990 to represent winter and summer conditions. $NH_4^+$ wet deposition and precipitation data were aggregated up to monthly or 4-week values for both CMAQ and NADP to calculate monthly $NH_4^+$ wet concentrations. The 4-week periods that were used for monthly values coincide with the beginning and end of the NADP collection time periods. The specific dates that represent January and June 1990 are January 9 to February 6, 1990 and June 5 to July 3, 1990, respectively. The 1990 period was used to leverage off a parallel CMAQ evaluation study that includes 1990 simulations.

Comparisons of daily and weekly collections in past studies have shown that a low bias exists in the NADP $NH_4^+$ data because the collection remains in the field for a week[23,24]. For this application, we accounted for a 15% $NH_4^+$ bias estimate in the weekly NADP data, similar to the average bias estimates based on multiple years of daily and weekly sampled data[23,24]. In addition, we are currently investigating the potential of a monthly or seasonal variation in this bias.

When merging the CMAQ and NADP data, points were removed from the analysis if fewer than 4 weeks of NADP data were available. Also, data points were removed from the analysis if the monitors were located within 9 grids (i.e., 324 km) of the western boundary of the model domain to remove boundary conditions from being a dominant influence.

## RESULTS

Figure 1 compares the January 1990 $NH_4^+$ wet concentrations between NADP-monitored data and CMAQ model simulations before and after emission adjustments. The adaptive-iterative DKF converged on a 64% decrease in emissions (i.e., 0.36 × annual $NH_3$ emissions) after three iterations. Note that the number of iterations is determined by the number of times the methodology must be applied until the emissions changes are $\Delta E \approx 0$, as previously described. Decreased $NH_3$ emissions reduced the large over-predictions while introducing an under-prediction at other monitors, with largest under-predictions located at monitors near the shoreline of the Great Lakes. The Root Mean Square Error (RMSE) = 0.36 mg/l before and RMS = 0.23 mg/l after the adjustment listed above. Based on a bias calculation for each monitor ((observation–predicted)/observation), the emission adjustment reduced the mean bias for all monitors from a 70% over-prediction to a –30% under-prediction. According to the RMSE and the bias calculation, the 64% decrease in emissions improved the model's simulation of $NH_4^+$ wet concentrations. The correlation coefficient (R) remained approximately the same before and after the emissions adjustment ($R_{before} = 0.44$ and $R_{after} = 0.45$). It is expected that R will not change significantly in this application because the spatial distribution of the emissions is not altered, so that the scatter is similar before and after the emission adjustments.

Being concerned about the under-predictions evident in Fig. 1, these data were spatially mapped to see if any regionally

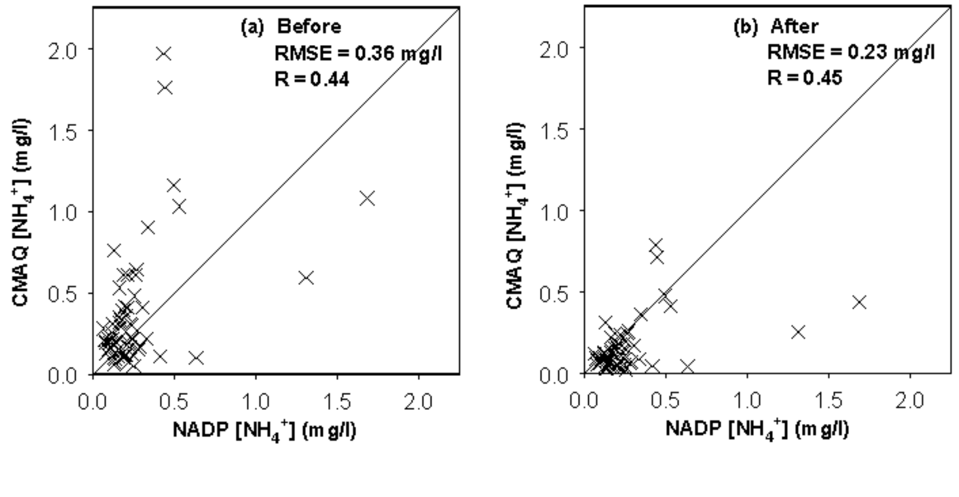

**FIGURE 1.** January 1990 (a) before and (b) after $NH_3$ emission adjustment.

coherent spatial biases existed before and after the decrease in emissions (Fig. 2). If so, this would suggest that the spatial distribution of the $NH_3$ emissions might have discrepancies. Many of the extreme model over-predictions were improved with the 64% decrease in $NH_3$ emissions (Fig. 2B). These over-predictions were in central states such as Illinois, Indiana, Kentucky, Tennessee, North Carolina, and Virginia, which are within a large area of $NH_3$ emissions from hog and cattle sources. However, in some northeastern states and the coastline of the Great Lakes, a prior under-prediction grew larger with the $NH_3$ emission decrease. If the same plots were shown using the absolute differences, the under-prediction bias in the Northeast would be less visible, since the concentrations in this area are relatively low. Since mobile emissions comprise more than 20% of the total $NH_3$ emissions in states including New Jersey, New Hampshire, Connecticut, Massachusetts, Maine, and New York, it is anticipated that this

A

B

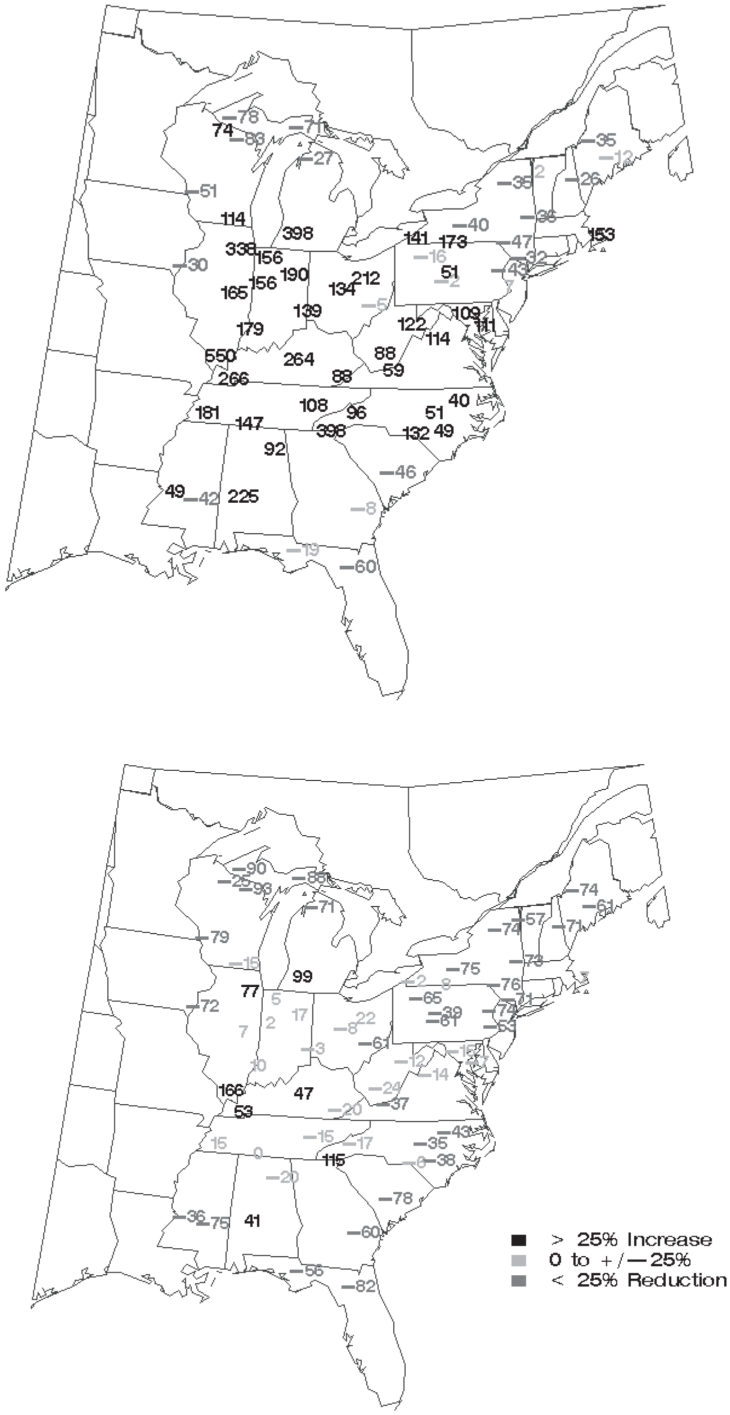

**FIGURE 2.** January 1990 % bias before (A) and after (B) emission adjustments ((Modeled-Observed)/Observed). Note that bias values greater than ±25% are shaded the same color.

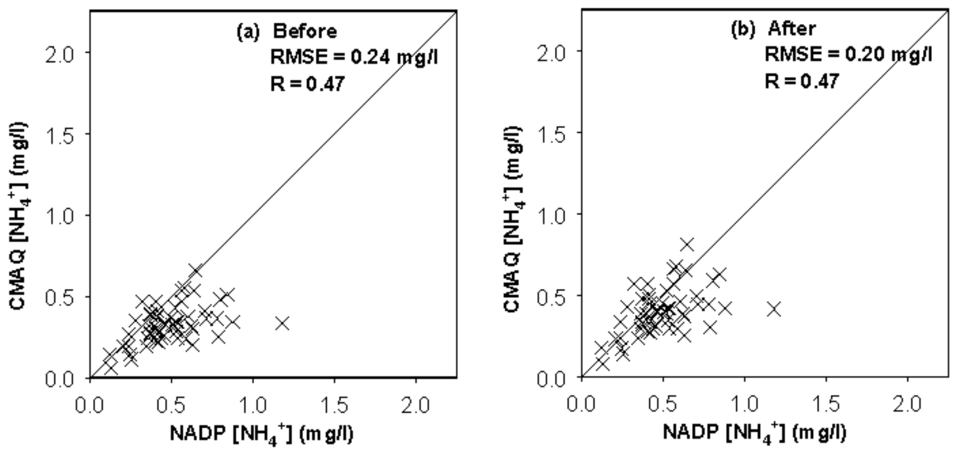

**FIGURE 3.** June1990 NADP vs. CMAQ $NH_4^+$ before and after $NH_3$ emission adjustments.

northeastern spatial bias may improve once the simulation is repeated using the newest NEI $NH_3$ inventory, which includes mobile emissions.

Figure 3 compares the June 1990 $NH_4^+$ wet concentrations between NADP monitored data and CMAQ model simulations before and after two iterations of the adaptive-iterative DKF, which converged on a 25% increase in emissions (i.e., 1.25 × annual $NH_3$ emissions). From the scatter plot, the simulation using the annual average $NH_3$ emissions has a clear tendency to under-predict $NH_4^+$, and this under-prediction is reduced after the inverse modeling adjustment to the emissions. The results after emission adjustments show a slight improvement to the RMSE and bias calculations. The RMSE = 0.24 mg/l before the emissions were adjusted, and RMSE = 0.20 mg/l after the adjustment listed above. The increase in emissions reduced the mean bias from an under-prediction of -20 to -11%. The correlation coefficient (R) remained the same before and after the emissions adjustment ($R_{before} = 0.47$ and $R_{after} = 0.47$).

Significantly varying spatial biases in $NH_4^+$ were not obvious in June 1990 (not shown). When the percentage bias between the CMAQ model and NADP data was considered before and after the 25% increase in $NH_3$ emissions, the comparison suggested that no regionally coherent spatial biases exist in the June 1990 case. Prior to the emission increase, a broad under-prediction bias was predominant over most of the domain and lessened after the increase in $NH_3$ emissions. This suggests fewer errors exist in the spatial distribution of $NH_3$ emissions for June 1990 as compared to January 1990. If so, the spatial distribution of $NH_3$ emissions could differ seasonally, which is also important information for the further development and refinement of $NH_3$ emission inventories.

## CONCLUSIONS

The adaptive-iterative DKF methodology is used herein to estimate seasonally varying $NH_3$ emission based on $NH_4^+$ wet concentration data from the CMAQ model and NADP. A decrease of 64% and an increase of 25% from the annual average values were estimated for January and June 1990, respectively. The RMSE and mean bias summary statistics suggested that these adjustments improved the results. A more rigorous comparison of independent data including ambient concentrations of sulfate and nitrate aerosols will also be included in this study to determine whether these $NH_3$ emission adjustments results in overall improvements. The seasonality suggested here supports the general findings of Pierce and Bender[5], where highest (lowest) emissions were estimated for the summer (winter) periods. Since the largest emission sources involve the volatilization of $NH_3$ from animal waste or fertilizer application, it is logical that emissions would be larger during higher temperatures typical of summer conditions than during colder winter conditions. More importantly, the results confirm that annual average emission fields can introduce substantial errors into air quality modeling results. If temperature is a dominant factor in determining the seasonal or temporal variability of the emissions, it suggests that meteorological conditions must be considered when developing $NH_3$ emission estimates for these models.

As a continuation of this study, $NH_3$ emission estimates will be produced for the spring and fall periods of 1990 to provide a complete seasonal cycle for analysis. Where available, we will also provide independent comparisons against other ambient data. If the $NH_3$ emission adjustments improve $NO_3$ and $NH_x$ concentrations as well as the $NH_4^+$ wet concentration data that were used in the inverse methodology, our confidence in the emission adjustments prescribed by the inverse modeling application will increase. These results will appear in a forthcoming paper.

## DISCLAIMER

This paper has been subjected to U.S. Environmental Protection Agency peer review and approved for publication. Mention of trade names or commercial products does not constitute endorsement or recommendation for use.

| | | |
|---|---|---|
| Received: | August | 29, 2001 |
| Revised: | October | 10, 2001 |
| Accepted: | October | 15, 2001 |
| Published: | November | 21, 2001 |

## BIOSKETCH

**Dr. Alice Gilliland** is a research scientist in the National Oceanic and Atmospheric Administration's Air Resources Laboratory, and is on assignment to the U.S. Environmental Protection Agency's Office of Research and Development. She is located in the Atmospheric Sciences Modeling Division in Research Triangle Park, North Carolina. Dr. Gilliland received her Ph.D. in Earth and Atmospheric Sciences from Georgia Institute of Technology. Her research interests include air quality modeling and evaluation, inverse modeling for source and sink estimation, and downscaling of global to regional scale modeling.
